# Optimizing Routine Malaria Surveillance Data in Urban Environments: A Case Study in Maputo City, Mozambique

**DOI:** 10.4269/ajtmh.22-0166

**Published:** 2022-10-03

**Authors:** Gillian Stresman, Ann-Sophie Stratil, Sergio Gomane, Sarmento Armando, Maria Rodrigues, Baltazar Candrinho, Arantxa Roca-Feltrer

**Affiliations:** ^1^ Department of Infection Biology, Faculty of Infectious and Tropical Diseases, London School of Hygiene & Tropical Medicine, London United Kingdom;; ^2^ Malaria Consortium, London, United Kingdom;; ^3^ Malaria Consortium, Maputo, Mozambique;; ^4^ National Malaria Control Program, Maputo, Mozambique

## Abstract

In urban settings in malaria-endemic countries, malaria incidence is not well characterized and assumed to be typically very low and consisting largely of imported infections. In such contexts, surveillance systems should adapt to ensure that data are of sufficient spatial and temporal resolution to inform appropriate programmatic interventions. The aim of this research was to 1) assess spatial and temporal trends in reported malaria cases in Maputo City, Mozambique, using an expanded case notification form and 2) to determine how malaria surveillance can be optimized to characterize the local epidemiological context, which can then be used to inform targeted entomological investigations and guide implementation of localized malaria responses. This study took place in all six health facilities of KaMavota District in Maputo City, Mozambique. A questionnaire was administered to all confirmed cases from November 2019 to August 2021. Households of cases were retrospectively geolocated using local landmarks as reference. Overall, 2,380 malaria cases were reported, with the majority being uncomplicated (97.7%) and a median age of 21 years; 70.8% of cases had reported traveling outside the city in the past month with nine reporting traveling internationally. Maps of the 1,314 malaria cases that were geolocated showed distinct spatial patterns. The expanded case notification form enables a more granular overview of the malaria epidemiology in Maputo City; the geolocation data clearly show the areas where endemic transmission is likely, thus informing where resources should be prioritized. As urbanization is rapidly increasing in malaria endemic areas, identifying systems and key variables to collect ensures an operational way to characterize urban malaria through optimization of routine data to inform decision-making.

## INTRODUCTION

A central pillar of the global malaria burden reduction strategy is using surveillance as a core intervention.^1^ The availability of robust epidemiological data can promote evidence-based decision-making so programs can effectively respond and adapt to changing trends, a critical component to successfully achieving malaria elimination.^2^ In many malaria-endemic countries, routine malaria surveillance consists of recording and reporting the number of malaria cases diagnosed per facility per month. In high transmission areas, the routine malaria surveillance data are able to capture broad spatial and temporal trends that reflect burden.^3^ However, as transmission intensity decreases and malaria becomes more heterogeneous, surveillance systems must adapt to ensure that the spatial and temporal resolution of malaria surveillance data reflects the microepidemiology.^4^ Knowing where to target resources within health facility catchment areas and having timely data on which to base decisions becomes increasingly relevant as transmission decreases to track progress toward achieving and maintaining malaria elimination.^5^

In urban settings in malaria-endemic countries, malaria incidence is not well characterized.^2^ The higher burden in rural areas has understandably diverted attention and resources away from characterizing urban malaria, and there has been an assumption that urban settings are largely “sinks” for malaria and the low burden observed in cities consists largely of imported infections.^6^ However, there is increasing evidence that malaria is transmitted in urban settings.^7^ Prevalence surveys that have been conducted in urban areas consistently identify a small percentage of infected individuals, and entomological surveys have found *Anopheles* mosquitoes in multiple African cities.^8^^,^^9^ Given the fast pace of urbanization, particularly in sub-Saharan Africa, and the changing climates, there is a growing concern of the impact on malaria transmission in urban environments.^10^^,^^11^ For countries to achieve malaria elimination, the question around the extent of malaria transmission in urban settings must be addressed. Surveillance systems should be in place that are appropriate to the low transmission, urban context that can effectively measure any spatiotemporal transmission dynamics so that programs can deploy adequate interventions, targeted to where they can be most impactful.^12^ Identifying how routine surveillance systems can be enhanced and the data used to inform and adapt surveillance system and any resulting programmatic activities is a current gap in most urban settings in sub-Saharan Africa.

Therefore, the aim of this study was to 1) assess spatial and temporal trends in reported malaria cases in Maputo City, Mozambique, using an expanded case notification form and 2) to determine how malaria surveillance can be optimized to characterize the local epidemiological context, which can then be used to inform targeted entomological investigations and guide implementation of localized malaria responses.

## METHODS

### Study area.

Mozambique is among the countries with the highest malaria burden, and transmission is heterogeneous. Maputo City, the capital of Mozambique, is located in the southern area of the country on the west side of Maputo Bay, with a climate alternating between a cool, dry season extending from approximately April through October and a hot rainy season from November through March each year.^13^ Malaria burden in Maputo City is low, with *Plasmodium falciparum* being the predominant species and an estimated prevalence in children aged 2 to 10 years of 5.2% (PfPR_2-10_) in 2019.^14^ Because Maputo City and Maputo Province have very low transmission, this urban region has been excluded from the mass distribution of long-lasting insecticide-treated bed nets (LLINs) in the eight of the 10 provinces with endemic malaria. The main vector-control intervention being implemented in Maputo Province is targeted indoor residual spraying, but this is not currently informed by case data. Maputo City includes seven districts, each of which is further subdivided into smaller city quarters or neighborhoods, referred to as Bairros. A rapid epidemiological descriptive assessment conducted by the malaria program in 2018 (unpublished) identified KaMavota as the district with the highest number of malaria cases according to health facility register books, accounting for almost 40% of all cases in the province. The health system in KaMavota District consists of six primary public facilities, excluding the general hospital in the area, that offer testing for malaria. Health facilities report the total number of confirmed cases per week using the Weekly Epidemiological Bulletin or per month using the district health information software, which is the backbone of the national malaria surveillance system to inform programmatic decision-making.

### Data collection.

This activity took place in the six primary health facilities of KaMavota District in Maputo City, Mozambique, where an expanded case notification form tailored to the urban environment was piloted as part of an initiative implemented by the National Malaria Control Program to improve surveillance in this urban district. All malaria cases reporting to the health facilities between November 2019 and August 2021 were administered a paper-based case notification form by trained health facility personnel to the patient or in the case of children, the parents or caregiver. Over the 2 years of data collection, information collected included age, sex, use of vector control interventions, and self-reported previous malaria infections, as well as any travel outside the city in the previous month, if traveled where they traveled to, and reasons for attending that specific facility. All patients were also asked to identify points of interest or unique landmarks, such as churches, schools, or shops, near their household to support programmatic mapping of cases. After data collection was completed, the records were double-entered using Excel-based forms by data clerks. Points of interest were located on freely available online maps (e.g., Google Maps, GeoNames.org), and points were digitized to represent the approximate location of the case household.

This study was implemented by the National Malaria Control Program to pilot an enhanced case report form to improve malaria surveillance. The data were collected as part of routine health services and therefore in the public domain, and no informed consent was sought. Because this was a secondary analysis of program data that were collected to support programmatic decision-making, the malaria control program classified this analysis as nonresearch and exempt from receiving ethical approval.

### Data analysis.

First, an exploratory analysis was conducted to assess trends in the data, including patterns of missingness to characterize the programmatic nature of this pilot data collection. Missing data were included as a separate category when summarizing the data or removed if required to implement the analysis.

#### Spatial analysis.

The subset of points that were successfully geolocated were compared with the full dataset to assess any potential selection bias. The case data were then summarized and visualized, including generating maps of case counts at multiple spatial scales: cases were aggregated to the health facility and neighborhood, also known as Bairro spatial resolution, and mapped to the point location to assess spatial trends. The trends and resulting inference associated with presenting the data at different spatial resolutions were compared to assess the advantages of collecting the location data according to the different spatial scales.

#### Regression analysis.

Logistic regression was used to assess any factors associated with a case reporting travel outside the city in the previous month and used as a proxy for infections that may have been imported and not acquired locally. Variables tested include age as a continuous variable, sex, occupation, reason for attending a specific clinic, reported use of vector control, and epidemiological week. On the basis of the results of the initial models, occupation was aggregated into categories of student, unemployed, or other and reason for attending into visiting, local to the area (whether living or working there), closest emergency service, and other. Generalized linear models, mixed effects, and generalized additive models were tested, and the Akaike information criterion was used to determine the best model fit. All analysis, including generating the maps, was conducted using R statistical software (Version 4.1.1).

## RESULTS

### Characterizing malaria cases.

There were 2,380 patients with confirmed malaria infections reported through case notification forms between November 2019 and August 2021. The use of paper-based questionnaires resulted in some missing data; however, no distinct patterns in the missingness were observed (Supplemental Figure 1). The highest malaria burden occurred during the summer months with the peak at epidemiological week 22, which coincides with the rainy season between April and October. The majority of malaria cases were uncomplicated, confirmed by rapid diagnostic test (RDT), and had not reported having a malaria infection in the previous month (Table 1). The median age of cases was 21 years (interquartile range = 8–34 years), 51.9% were male, and 78.5% of patients reported using at least one form of vector control to protect against malaria, with the use of LLINs being the most common. The predominant occupation category reported by cases was being a child (i.e., too young to be relevant and not yet in school; 32.8%), a student (18.3%), or other (26.3%). The majority (73.7%) of patients reported that the reason they visited a specific health facility was because they lived close by (Table 2).

**Table 1:** Summary of the variables on malaria and clinical care-seeking in all cases reported during the study period and the subset that were successfully geolocated to the household

	All cases (*N* = 2,380)		Subset with latitude/longitude (*n* = 1,314)	
Variables	Values	No. missing	Values	No. missing
Epidemiological week, median (range)	22 (1–53)	179	25 (1–53)	52
Notification date, median (IQR)	July 31, 2020 (Mar. 27, 2020–Jan. 16, 2021)	30	Oct. 12, 2020 (June 8, 2020–Feb. 3, 2021)	40
Uncomplicated malaria diagnosis, % (*n*)	97.1 (2,074)	244	97.6 (1,168)	118
Malaria treatment in past 1 month, % (*n*)	5.5 (117)	269	6.2 (73)	135
Notifying profession, % (*n*)				
Doctor	5.6 (113)	93	3.9 (50)	43
Nurse	35.8 (818)		41.2 (524)	
MHC nurse	12.5 (287)		13.7 (174)	
Curative technician	37.3 (853)		33.1 (421)	
Preventive technician	1.9 (44)		0.2 (3)	
Other	6.6 (152)		7.8 (99)	
Notifying ward, % (n)				
Adult	55.5 (1,280)	76	54.1 (695)	29
Pediatric	32.2 (744)		32.2 (414)	
Antenatal clinic	1.3 (30)		1.5 (19)	
Emergency department	8.0 (185)		8.4 (108)	
Other	2.8 (65)		3.8 (49)	
Malaria diagnostic method				
RDT	97.7 (2,268)	58	97.7 (1,256)	28
Microscopy	2.2 (51)		2.2 (28)	
None	0.1 (3)		0.1 (2)	

IQR = interquartile range; MHC = maternal health center; RDT = rapid diagnostic test. The value of each variable and the number of cases in which that specific variable was missing are shown. Unless indicated, the number in parenthesis represents the denominator used to calculate each percentage.

**Table 2:** Summary of demographic information for all cases reported during the study period as well as the subset that were successfully geolocated to the household

	All cases (*N* = 2,380)		Subset with latitude/longitude (*n* = 1,314)	
Demographics	Values	No. missing	Values	No. missing
Age, median (IQR)	21 (8–34)	50	21 (7–34)	20
Sex, % male (*n*)	51.9 (1,190)	88	49.4 (622)	55
Occupation group				
Miner	0.1 (3)	378	0.3 (3)	212
Farmer	5.0 (101)		3.8 (42)	
Driver	2.3 (47)		2.5 (28)	
Student	18.3 (368)		15.8 (174)	
Fisherman	0.6 (13)		0.3 (3)	
Unemployed	13.1 (263)		12.9 (142)	
Community worker	1.2 (25)		1.7 (19)	
Child	32.8 (658)		36.8 (406)	
Other	26.3 (524)		25.9 (285)	
Traveled past month, % yes (n)	70.8 (1,927)	453	75.7 (830)	217
If traveled, duration in days, median (IQR)	13 (6–27)	656	13 (6–28)	427
Use of vector control, % yes (n)	78.5 (1,613)	767	77.1 (652)	469
If use vector control, which type?				
IRS	9.4 (109)	108	3.2 (19)	62
LLIN	86.2 (998)		91.7 (541)	
Other	4.4 (51)		5.1 (30)	
Why did you visit this visit this facility?				
Emergency department services	9.1 (153)	705	8.4 (78)	388
Live close	73.7 (1,235)		77.5 (718)	
Work close	4.7 (79)		3.7 (34)	
Visiting the area	9.8 (164)		8.4 (78)	
Other	2.6 (44)		1.9 (18)	

IQR = interquartile range; IRS = indoor residual spraying; LLIN = long-lasting insecticide-treated bed nets. The value of each variable as well as the number of cases in which that specific variable was missing is shown. Unless indicated, the number in parenthesis represents the denominator used to calculate each percentage.

### Malaria cases reporting recent travel.

**Figure 1.**
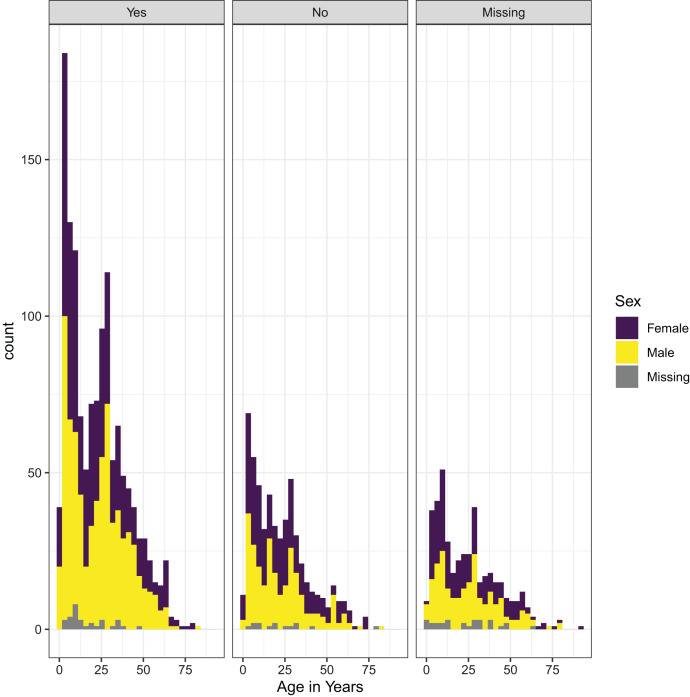
Histograms showing the age distribution of malaria cases presenting for care during the study period according to recent travel. Each panel shows the differences according to whether the case reported recent travel (No, Yes), including those in which the response to this question was missing. The age trends in each panel are colored to show the proportion in each age that are females (purple, top shade), males (yellow, middle shade), or where sex was missing (gray, bottom shade).

**Figure 2.**
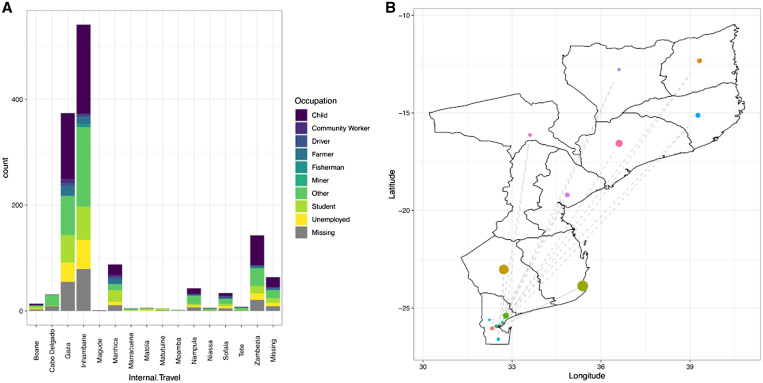
Characteristics of malaria cases that reported having traveled within Mozambique in the previous 4 weeks. (**A**) Bar graph of the location of reported travel, with each bar divided by the reported occupation of the case, including where the response was missing. (**B**) A map of Mozambique showing the destination of where cases reported to have traveled, with the size of each circle reflecting the relative number of cases per destination.

Of all the cases, 70.8% reported having traveled recently and may have represented infections imported into the city. Interestingly, the age distribution of cases was similar, regardless of recent reported travel (Figure 1). Of note is that the majority of cases were observed in young children and working-age adults (Figure 1). The lack of age pattern was also observed when adjusting for factors associated with cases that traveled or not. Specifically, when accounting for the seasonal trend of malaria transmission as the smoothed term in the generalized additive models, malaria cases that reported being students (–0.664, 95% confidence interval [CI]: –1.074 to –0.096) or unemployed (–0.663, 95% CI: –1.261 to –0.193) were less likely to have traveled (Table 3). The majority of travelers visited other locations within Mozambique, and only nine reported having traveled internationally. Of those who traveled within the country, the majority reported traveling to Gaza and Inhambane where in 2018 there was an estimated RDT prevalence in children 6 to 59 months of age of 16.9% and 35.1%, respectively.^15^ Similar to the dataset with all confirmed malaria cases, including those where spatial coordinates were not available, children and those classified as “other” were reported as being the predominant occupation groups (Figure 2). No differences were observed in cases reporting recent travel during the high and low malaria transmission seasons, the destination reported, or the relative number of travelers visiting the different locations (Supplemental Figure 2).

**Table 3:** Results of the general additive model for factors associated with cases that reported traveling in the previous 4 weeks

Linear predictors	Beta	95% CI	*P* value
Intercept	0.521	−1.821 to 2.893	0.663
Occupation group			
Other	1	–	–
Student	−0.664	−1.074 to −0.096	0.008
Unemployed	−0.663	−1.261 to −0.193	0.017
Reason for attending			
Local	1	–	–
Visiting	0.22	−0.514 to 0.991	0.564
Other	−0.06	−1.347 to 1.256	0.924
Use vector control	0.015	−0.499 to 0.466	0.952

Smooth terms	edf	Standard error	*P* value
Health facility	21.63	5.573	0.0003
Epidemiological week	2.50	0.026	< 0.001

CI = confidence interval; edf = effective degrees of freedom (degree of non-linearity in the modeled variable). The linear predictors as well as the smoothed terms for health facility and epidemiological week are presented.

### Spatial trends in subset of malaria cases geolocated.

In total, 1,314 (55.2%) of the malaria cases were successfully geolocated and mapped to the reported point of interest. However, the trends in the clinical data were similar between all cases, and the subset that was successfully geolocated (Table 1). In the geolocated subset, the median week of case presentation was later, but otherwise the populations are similar. The demographic trends in the subset of cases for which spatial coordinates were available were also similar to those in the full dataset. The main exception was that more cases reported traveling recently in the geolocated subset, with 75.7% of cases (compared with 70.8% in the full case dataset) (Table 2).

**Figure 3.**
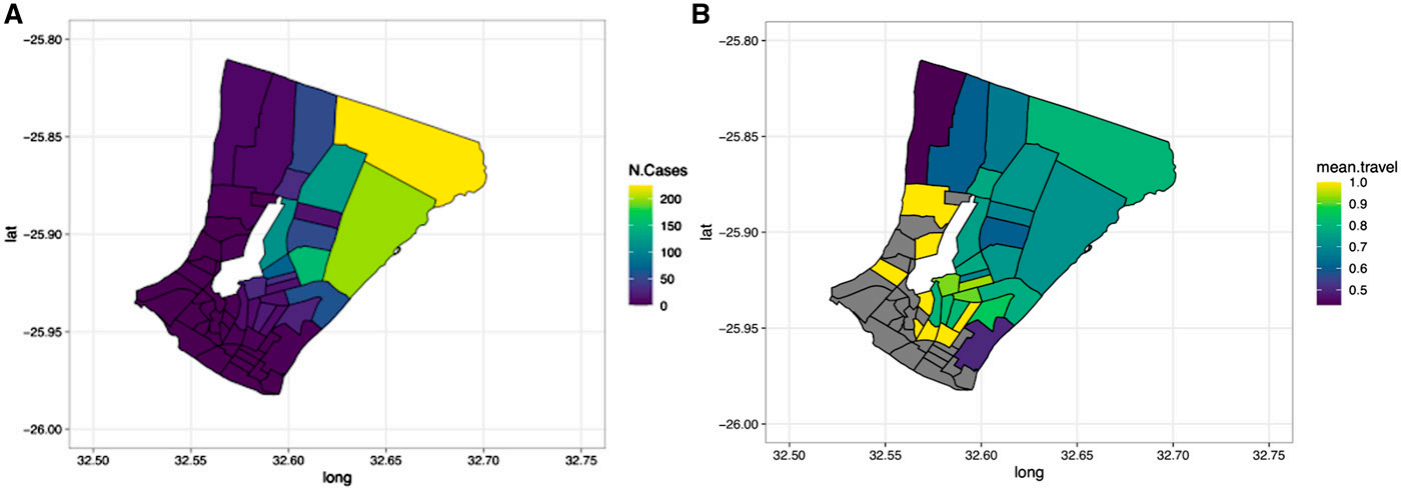
Maps of KaMavota District in Maputo City with each Bairro shown by the black boundaries. The number of cases (**A**) and the proportion of cases reporting traveling within the previous 4 weeks (**B**) are represented by the different colors of each Bairro. Bairros in gray represent those where no cases reported having traveled in the previous 4 weeks.

Maps of burden, or the number of malaria cases at the Bairro level, showed cases distributed across most of the city, but there was a higher burden of malaria in the northeastern Bairros (Figure 3A). The burden in the northeastern part of the city was consistently higher, relative to the rest of the Bairros in the study area across the transmission season (Supplemental Figure 3). When comparing the spatial distribution of malaria burden with the proportion of the cases that reported recently traveling, a different pattern was evident. For example, in Bairros in the southwestern part of the city, all malaria cases that were geolocated to that area reported traveling within the past month, and thus there may not be significant local transmission in the area (Figure 3B). Within the subset of cases that were geolocated, there was less temporal variability in cases per Bairro (Supplemental Figure 3).

**Figure 4.**
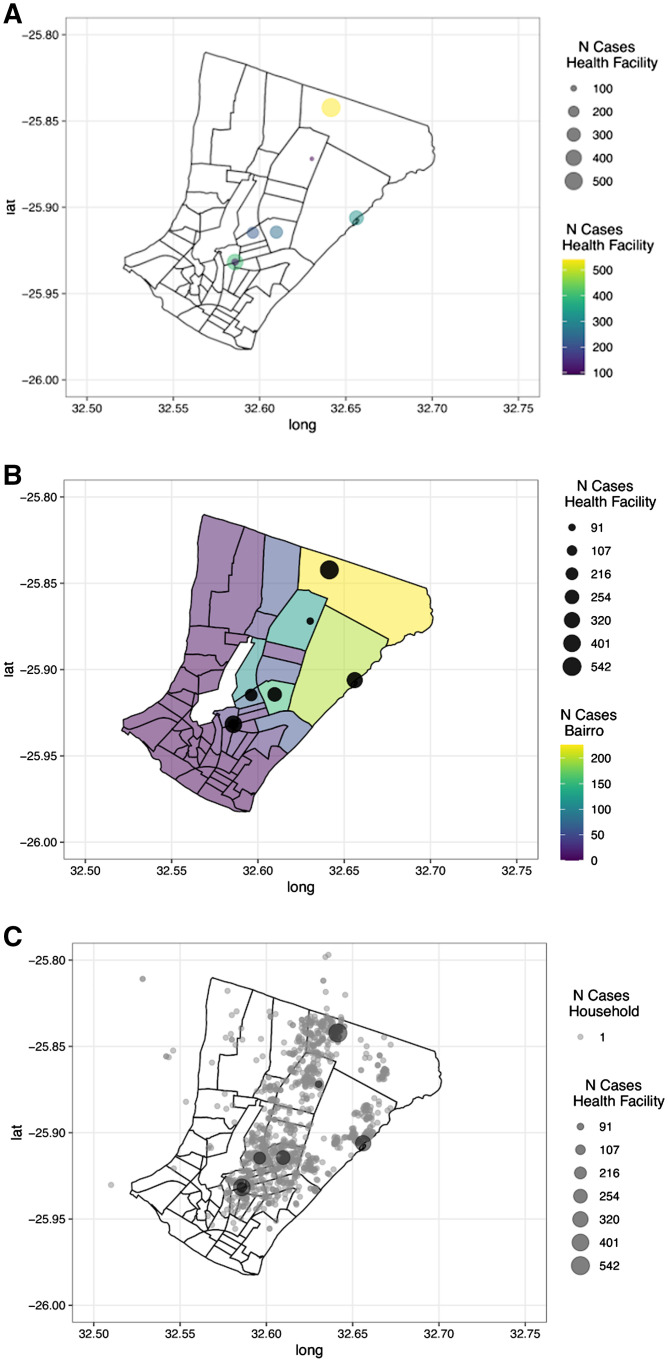
Collecting more granular spatial information of residence of cases increases the analytical power to observe trends in the subcatchment area heterogeneity of malaria. (**A**) Location of the health facilities, with both the size and color representing the number of cases reported per facility. (**B**) Location of the health facilities (black dots) with the size of each dot reflecting the number of cases reported with the color of each Bairro representing the number of cases reported that resided in each neighborhood. (**C**) Location of the health facilities (larger black dots), with the size reflecting the number of cases with the approximate location of each case (small dots) based on the household geolocation.

Finally, to assess the additional information gained by geolocating cases to the household, we mapped malaria burden at varying degrees of spatial resolution. Maps of malaria cases based on the location of the health facility show high burden in the northeastern area but also that there is a reasonable burden in the rest of the city despite being in Bairros where few malaria cases were reported (Figure 4A). Collecting information on the Bairro of residence, or relevant local administrative unit, would facilitate mapping a more nuanced picture of malaria heterogeneity within the city while being more operationally tractable compared with recording the location of the household or proxy based on local landmarks (Figure 4B). The high-burden area in the northeast is clearly visible, but the relatively uniform distribution of the cases across the rest of the city is apparent. The most granular option is to collect the approximate location of the household for confirmed cases (Figure 4C). Increasing the spatial resolution to the household clearly shows where malaria cases cluster in the city. However, for more robust analysis of these data, obtaining the precise location of the household, instead of assigning the location to a landmark close to the house would be required. Additionally, without spatial information on those testing negative for malaria or other nonmalarial illness, the areas without any confirmed cases could also reflect areas where populations may not be represented by the surveillance data.

## DISCUSSION

In this case study, we present demographic and spatial trends in 2,380 malaria cases presenting for care in KaMavota District of Maputo City, Mozambique. Here, transmission is consistent with other low-endemic settings with a broad age range of clinical malaria infections (as opposed to young children in high-endemic settings), and cases were highest during the months that coincide with the rainy season.^16^^,^^17^ The majority of cases were diagnosed by RDT and had not had a malaria infection in the previous month, and the reported use of vector control was high.

Critically, 30% of cases reported not having traveled recently, indicating that endemic transmission in this urban environment is likely. Therefore, malaria programs should target activities to determine the proportion of endemic infections that are seeded by imported infections so appropriate transmission reduction strategies (e.g., prophylaxis in travelers, targeted vector control) can be implemented accordingly. Furthermore, the majority of malaria cases that reported traveling recently had traveled to other parts of Mozambique, highlighting that in high-burden countries, unsurprisingly, imported infections into urban areas, are more likely to come from internal movement within national borders. In this population, children also traveled more frequently than was expected. The findings from other studies are that adults make up more of the population that travels as they seek work and children stay home.^18^ However, in this study, the destinations most reported by travelers are popular holiday destinations, so the high proportion of children traveling may be due to family holidays. Therefore, in this population, children are not a good proxy for ascertaining whether transmission in an area is likely endemic.

Maps of the 1,314 cases that were successfully geolocated showed distinct spatial patterns within the city. Even after accounting for the proportion of cases that traveled, the additional geolocation data clearly showed the areas in the city where endemic transmission is likely thus informing where more detailed entomological investigations and interventions should be prioritized. Conversely, when the spatial information on residence and travel history were combined, distinct areas of the city where cases are all likely imported from outside the city were detected.

This is the first attempt in Mozambique to introduce enhanced routine case data procedures. The additional data collection as part of routine practice proved to be feasible in this setting when the weekly number of malaria cases was low, meaning there was sufficient time to complete the case notification form. Despite the missingness in the data generated, it enabled a better characterization of malaria epidemiology in the urban setting of Maputo.

The more granular data collected as part of this enhanced surveillance protocol provided important insight into the microepidemiology of malaria in this urban setting. Household retrospective geolocation activity was not perfect with both respondents’ not being able to identify a point of interest or that point of interest not located on the map. However, it enabled a more granular assessment of malaria patterns in the city. Moving forward, the resolution of data to collect will depend on the intended use of those data. The household location is the most precise to create a map of malaria risk but requires additional analytical steps to convert the point data into inferences about malaria risk.^19^ Without a formalized address system, if the point-level data are not used to inform programmatic decision-making, the additional effort to collect these data may not be worthwhile. In contrast, collecting information on the Bairro, or smallest administrative unit in the city, may provide a simple way to increase the spatial resolution of the case data to delineate areas within health facility catchment areas with higher malaria burden to inform targeting of effort to these more granular populations. When transmission becomes low and more spatially heterogenous targeting interventions to the smallest spatial scale becomes a more efficient use of resources for maximum impact.^20^ However, the optimal spatial resolution of data to collect will depend on the programmatic priorities. When transmission is very low, household-level information becomes important as part of focus investigations to target residual transmission and confirm absence of transmission.^21^

Furthermore, the additional data collected provided a more detailed overview of the malaria epidemiology in Maputo City. To enhance this malaria surveillance platform further, use of an electronic data capture system recording information on each case would facilitate both the quality of the data and support built-in real-time analytical and data visualization functions and ensure that malaria programs can quickly respond and adapt to changing malaria trends.^22^ For such an individual-level data collection platform to be successful, key indicators and outputs should be agreed on and tested to ensure refinement of the collection tools before further rollout. Experiences gained from the programmatic introduction of the paper-based case notification form have informed the National Malaria Control Program’s digitalization efforts in the country, in which electronic case notifications are being applied in very low transmission settings. Defined thresholds for when switching from collecting aggregated data to individual-level data (e.g., in high-transmission settings, individual-level data collection on malaria may be less relevant) and what trends or intensity of malaria cases warrant a programmatic response would help translate the surveillance data to action.

The study has some limitations to note. First, this characterization of the urban malaria epidemiology was based on routine surveillance data. By definition, passive surveillance does not account for non–care-seeking infections, and the current system does not capture cases confirmed in nongovernmental facilities (e.g., private, nongovernmental) and thus may underrepresent the true scale of the malaria burden.^23^ However, our intention was to characterize the micro-epidemiological trends in malaria cases that can be learned from case data and not to quantify malaria infections in the city. Next, there was a high degree of missingness in the data collected. The use of paper-based forms precluded built-in data checks to minimize missing data, and geolocation of households was successful in slightly more than half of cases. However, except for the proportion of cases reporting travel between the full and subset that were successfully geolocated, we expect the missingness to be nondifferential and have minimal implications on the overall findings and still support useful programmatic insight. Other approaches, including data imputation could have been used; however, because the aim of this work was to support programmatic decision-making, we focused on analytical approaches that would be programmatically relevant. In addition, “other” categories were included with several categorical variables, but no open-ended options to specify were provided to simplify the data collection. This resulted in some questions having a sizeable proportion of responses as “other,” and it was not possible to further understand the trends. Finally, the travel patterns in malaria cases observed in this study include the period of the COVID-19 pandemic, which likely affected the degree of both care-seeking for malaria and international travel when international borders were closed. The implications could be that the prevalence of domestic travel may be higher than expected but would not change the overall finding that many cases are imported into the city from elsewhere in the country. Similarly, how the trends in cases who have traveled or not reflect trends in the broader population is unknown. It is possible that the Bairros with a high proportion of cases reporting travel could have other unmeasured factors that may influence the trends (e.g., socioeconomic status or occupational factors driving people to leave the city). Without additional data on the distribution of the general population either from a census or people attending the clinics for nonmalaria reasons, the trends cannot be fully elucidated. However, given that our aim was to identify areas within the city where endemic transmission may be occurring and thus triggering a more detailed assessment, the results are still meaningful from a programmatic context.

Routine malaria surveillance data are an imperfect but valuable source of data with which to characterize trends and on which to base decision-making to further reduce transmission. In Maputo or other urban areas where malaria transmission is low, adapting the case report form currently being used as part of routine malaria surveillance to include additional variables, particularly information on recent travel and the location of the household of cases using a digital platform, programs can gain valuable insight for decision-making. Furthermore, if geolocation information is collected routinely for those testing negative for malaria and/or other illnesses, malaria results could be interpreted within the context of the broader population distribution, strengthening the inferences that can be made. Such an enhanced surveillance system could identify areas where local transmission is likely for targeting entomological surveillance or focal interventions as well as monitoring trends to adapt quickly as the context changes. As urbanization is rapidly increasing in malaria-endemic areas and the climate is changing, understanding the malaria epidemiology in such environments is important to ensure that interventions and surveillance systems are adequate to respond to the unique risks in these urban settings. The results from this study may be useful for countries considering approaches to characterize urban malaria and target interventions through the optimization of routine data to inform decision-making.

## Supplemental files


Supplemental materials
