# Supplementary material for: Optimizing Routine Malaria Surveillance Data in Urban Environments: A Case Study in Maputo City, Mozambique
Source: Am J Trop Med Hyg. 2022 Oct 3;108(2 Suppl):24–31. doi: 10.4269/ajtmh.22-0166 (PMC9904158; doi:10.4269/ajtmh.22-0166)
Supplement: Supplementary file 1 [file tpmd220166.SD1.pdf]

Supplemental Figure Legends

Suppl Figure 1 – Frequency of missing data in some of the variables collected as part of the enhanced malaria surveillance program

Suppl Figure 2 – Location of reported internal travel, stratified by those who traveled during the high (A) and low (B) malaria transmission seasons. Here the high malaria transmission season was defined as those traveling between October to January each year to reflect the differences in malaria transmission patterns in the northern compared to the southern areas of the country.

Suppl Figure 3 – Number of cases by Bairro per month in people reporting no recent travel (A) and reporting travel within the past month (B)

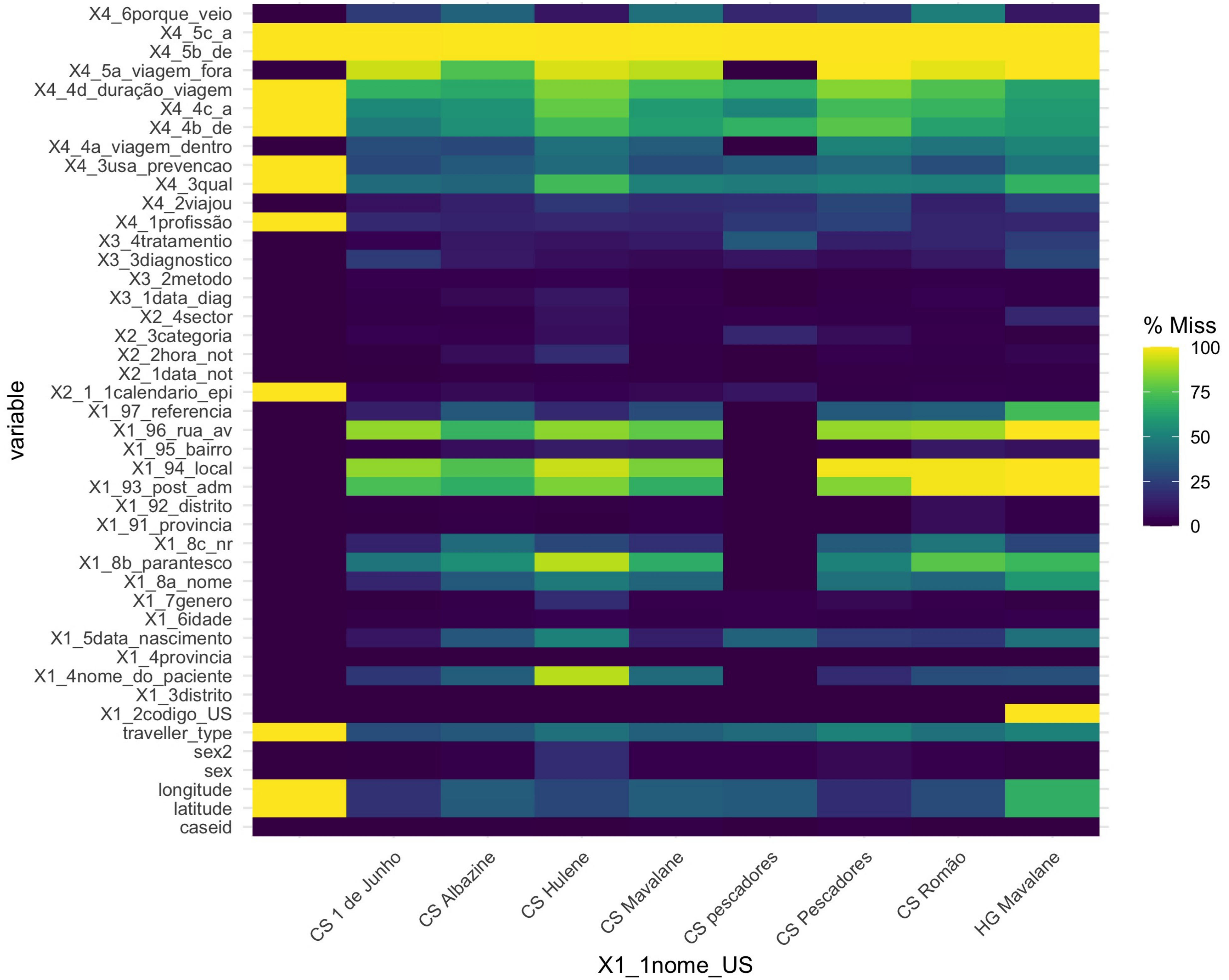
X4_6porque_veio
X4_5c_a
X4_5b_de
X4_5a_viagem_fora
X4_4d_duração_viagem
X4_4c_a
X4_4b_de
X4_4a_viagem_dentro
X4_3usa_prevencao
X4_3qual
X4_2viajou
X4_1profissão
X3_4tratamentio
X3_3diagnostico
X3_2metodo
X3_1data_diag
X2_4sector
X2_3categoria
X2_2hora_not
X2_1data_not
X2_1_1calendario_epi
X1_97_referencia
X1_96_rua_av
X1_95_bairro
X1_94_local
X1_93_post_adm
X1_92_distrito
X1_91_provincia
X1_8c_nr
X1_8b_parantesco
X1_8a_nome
X1_7genero
X1_6idade
X1_5data_nascimento
X1_4provincia
X1_4nome_do_paciente
X1_3distrito
X1_2codigo_US
traveller_type
sex2
sex
longitude
latitude
caseid
variable
CS 1 de Junho
CS Albazine
CS Hulene
CS Mavalane
CS pescadores
CS Pescadores
CS Romão
HG Mavalane
X1_1nome_US
% Miss
100
75
50
25
0

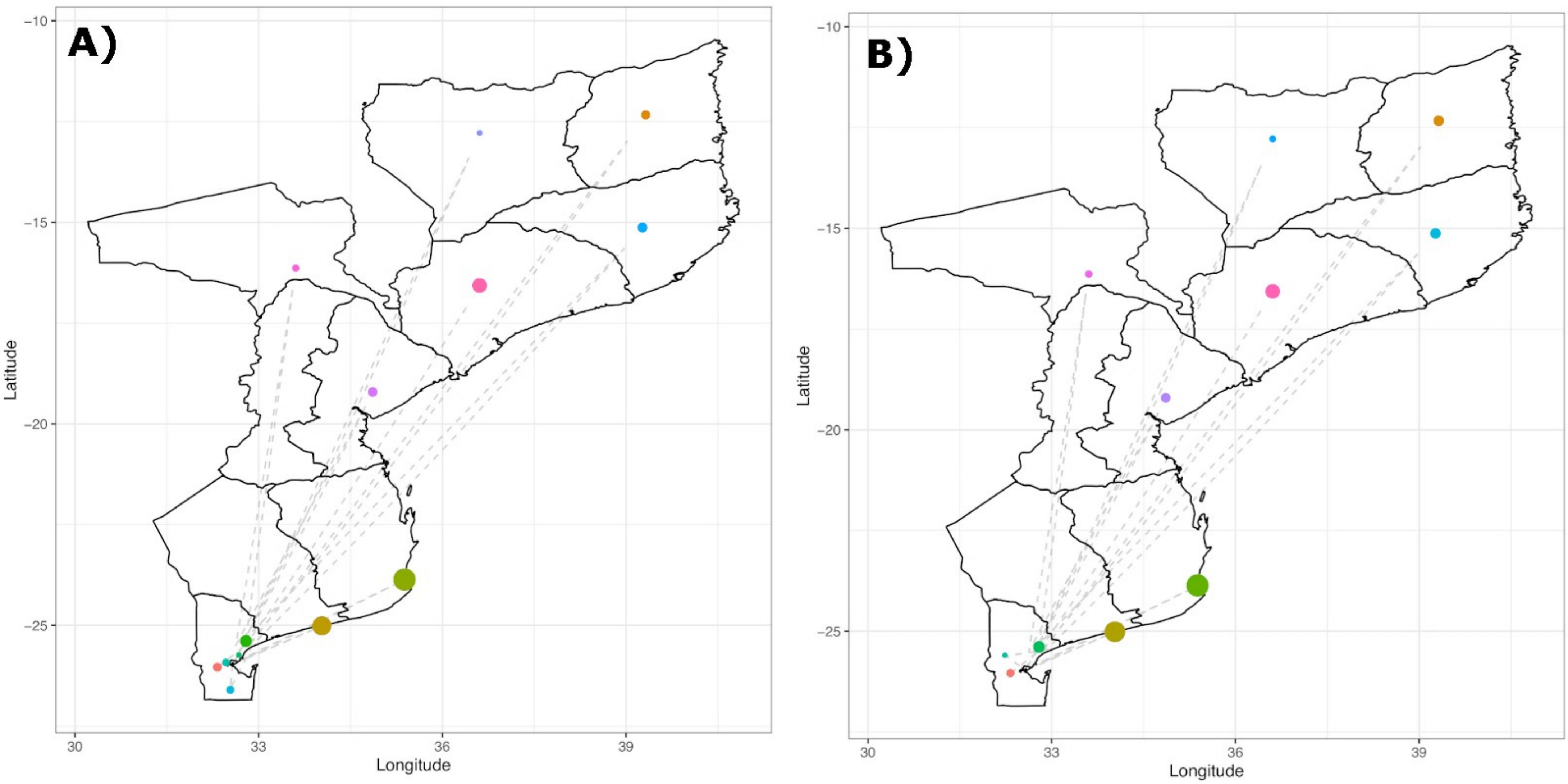

A)
B)
Latitude
Longitude
-10
-15
-20
-25
30
33
36
39

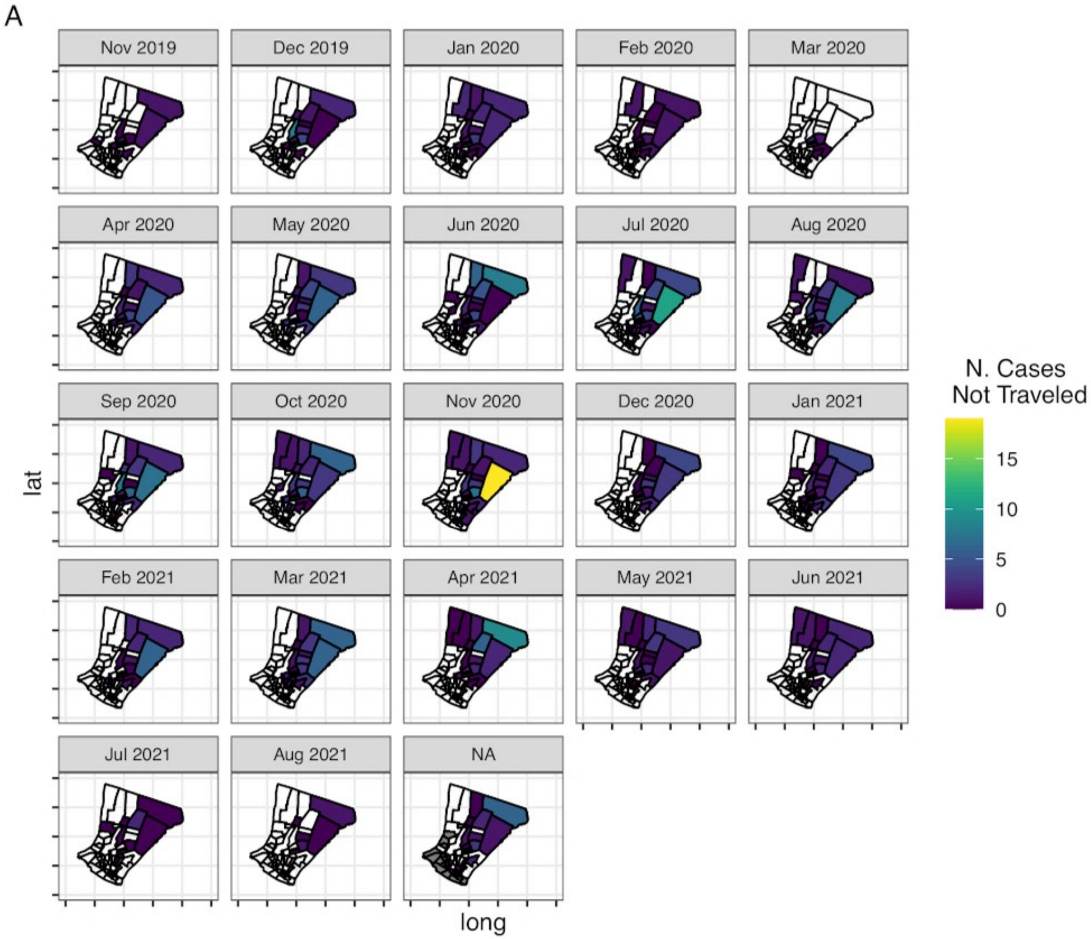
A
Nov 2019
Dec 2019
Jan 2020
Feb 2020
Mar 2020
Apr 2020
May 2020
Jun 2020
Jul 2020
Aug 2020
Sep 2020
Oct 2020
Nov 2020
Dec 2020
Jan 2021
Feb 2021
Mar 2021
Apr 2021
May 2021
Jun 2021
Jul 2021
Aug 2021
NA
lat
long
N. Cases
Not Traveled
15
10
5
0

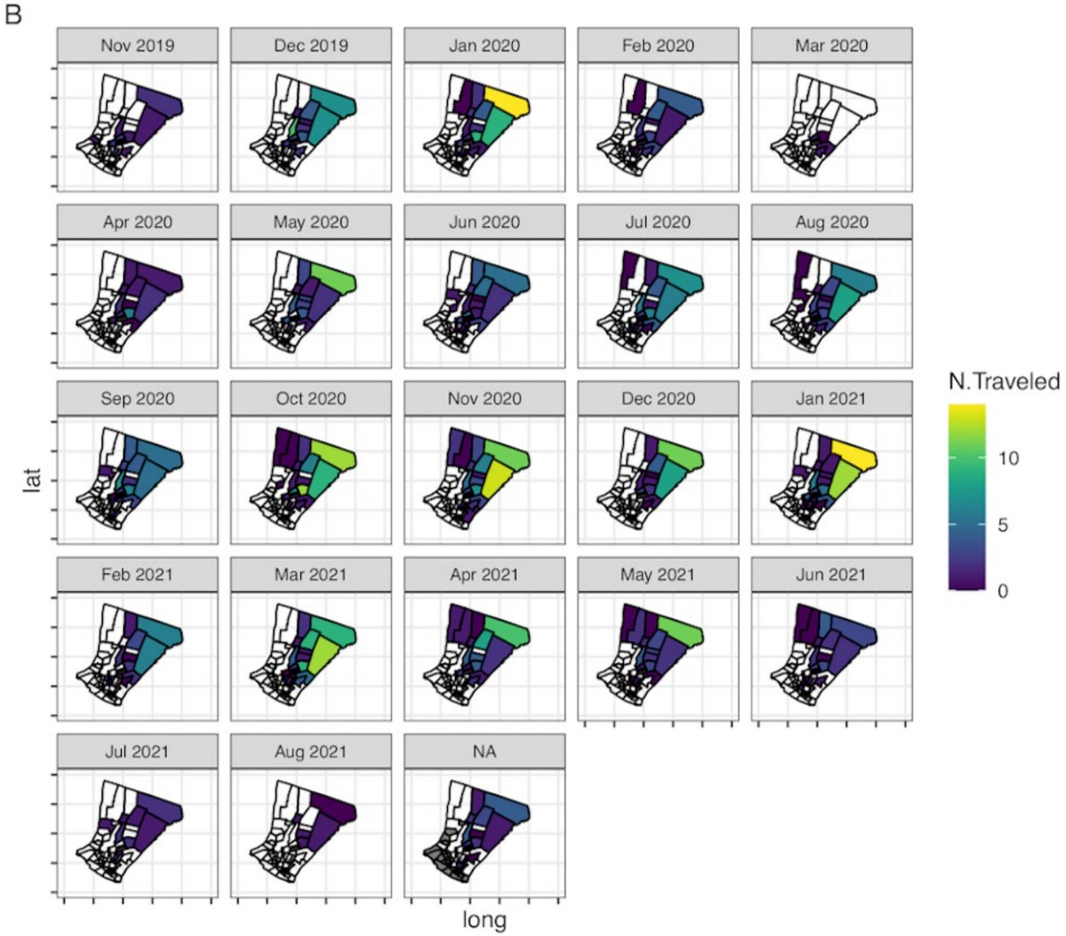
B
Nov 2019
Dec 2019
Jan 2020
Feb 2020
Mar 2020
Apr 2020
May 2020
Jun 2020
Jul 2020
Aug 2020
Sep 2020
Oct 2020
Nov 2020
Dec 2020
Jan 2021
Feb 2021
Mar 2021
Apr 2021
May 2021
Jun 2021
Jul 2021
Aug 2021
NA
lat
long
N.Traveled
10
5
0
